# A comprehensive review of detection, optical character recognition, and structuring methods for herbarium label digitization

**DOI:** 10.1002/aps3.70084

**Published:** 2026-11-06

**Authors:** Hiba Abbad, Hanane Ariouat, Nicolas Turenne, Eric Chenin, Abderrazak Sebaa, Edi Prifti, Jean‐Daniel Zucker, Youcef Sklab

**Affiliations:** ^1^ Laboratoire LITAN, École Supérieure en Sciences et Technologies de l'Informatique et du Numérique (ESTIN) Béjaïa Algeria; ^2^ Institut de Recherche pour le Développement (IRD) Sorbonne Université, UMMISCO Paris France; ^3^ INRAE MathNum France; ^4^ Muséum national d'Histoire naturelle (MNHN), Institut de Systématique, Évolution, Biodiversité (ISYEB) Paris France; ^5^ Sorbonne Université, INSERM, Nutrition et Obesities; systemic approaches, NutriOmique, AP‐HP, Hôpital Pitié‐Salpêtrière Paris France

**Keywords:** deep learning, herbarium digitization, label detection, large language models (LLMs), metadata structuring, named entity recognition (NER), optical character recognition (OCR)

## Abstract

Global herbarium digitization has produced over 50 million specimen images, many of which contain handwritten or printed labels with rich metadata such as species names, collection dates, localities, and ecological notes. Manual transcription of this information is labor intensive, error prone, and not scalable, which has motivated the development of automated solutions. This systematic review examines studies published between 2018 and 2026, identified through electronic database searches and backward reference screening, focusing on deep learning and computer vision approaches for herbarium label digitization. The reviewed methods include convolutional neural networks, transformer‐based models, large language models, and open‐source pipelines for optical character recognition and structured metadata extraction from historical and handwritten botanical records. The results highlight significant advances in label detection, text recognition accuracy, and metadata structuring enabled by deep learning techniques, although reported performance varies considerably depending on dataset quality, handwriting complexity, and domain adaptation strategies. Despite this progress, substantial challenges remain in handling historical handwriting styles, heterogeneous label layouts, and multilingual collections. Future research should emphasize the creation of curated benchmark datasets and the development of scalable, robust artificial intelligence (AI) pipelines to enhance the reliability, efficiency, and generalizability of automated herbarium digitization systems.

Digitizing physical records is essential for enabling large‐scale access to scientific and historical data, particularly in biodiversity research. Herbarium sheets are invaluable resources, often annotated with labels containing critical metadata such as species name, collection date, location, and ecological notes, which support applications including species distribution modeling, habitat analysis, and phenology studies (Willis et al., 2017; Nelson and Ellis, 2019), and are increasingly recognized as major sources of functional trait and phenological information at unprecedented spatial and temporal scales (Lorieul et al., 2019; Heberling, 2022). Although initiatives like the Global Biodiversity Information Facility (GBIF) and Integrated Digitized Biocollections (iDigBio) have made millions of specimen images publicly accessible (Yesson et al., 2007; GBIF, 2025; iDigBio, 2025), much of the associated label information remains unextracted, limiting the full scientific potential of these collections.

Manual transcription has historically served as the primary method for converting label information into digital form. While effective for small‐scale projects, it is time consuming, prone to inconsistencies, and unsustainable for large collections (Groom et al., 2019; Engledow, 2022). The rapid growth of digitization initiatives and the increasing diversity of herbarium collections have further highlighted the need for scalable, automated solutions capable of handling diverse label formats and specimen conditions (James et al., 2018; Ariouat et al., 2024, 2025; Sklab et al., 2025a, 2025b, 2025c). Herbarium labels present domain‐specific challenges that complicate automated extraction. Labels vary widely in layout, language, font style, and handwriting, and they frequently appear alongside barcodes, stamps, and plant specimens on the same sheet, creating visually complex scenes (Figure 1). This heterogeneity reduces the performance of traditional optical character recognition (OCR) pipelines and increases the difficulty of detecting and segmenting relevant text regions (Drinkwater et al., 2014).

**Figure 1:**
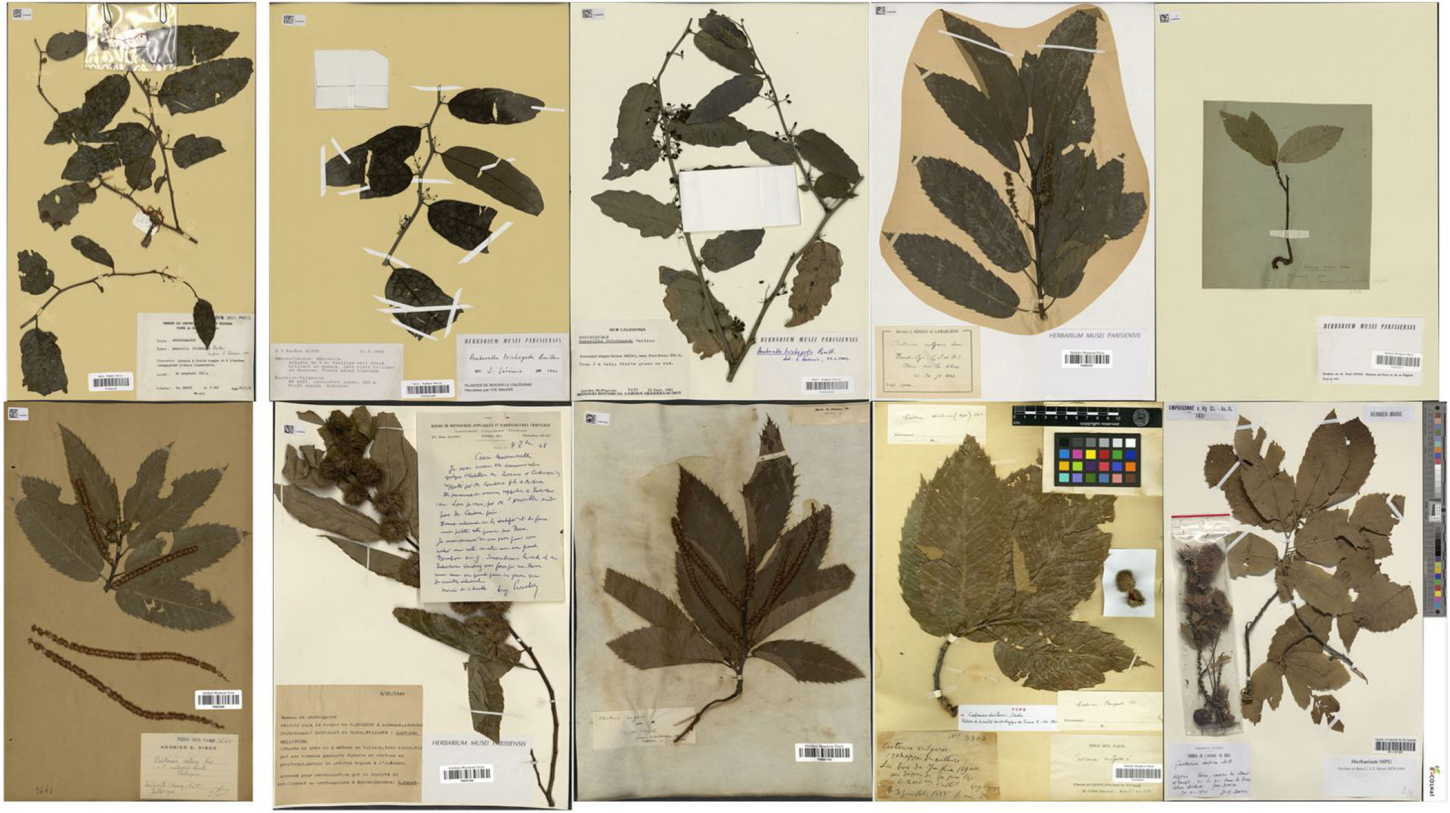
Examples of herbarium sheets showing label diversity in format, handwriting, and layout, often mixed with plant material and stamps. Reproduced from Ariouat et al. (2025) (CC BY‐NC‐ND).

Recent advances in computer vision and natural language processing, particularly deep learning–based OCR, transformer architectures, and large language models, have enabled promising progress in automated label extraction. However, existing reviews have not provided a focused synthesis of label transcription methods and dataset‐specific constraints. Hussein et al. (2021) and Tessler and Little (2026) primarily address broader aspects of herbarium digitization, while Guo et al. (2025), despite offering a comprehensive review of artificial intelligence (AI) methodologies for herbarium specimen image analysis, cover classification, segmentation, and multimodal frameworks rather than the label processing pipeline specifically. Furthermore, the lack of standardized benchmarking and comparable performance metrics across studies reinforces the need for a dedicated review of this domain (Soltis, 2017; James et al., 2018). The present review addresses these gaps by concentrating on the three sequential stages of label processing: detection, OCR, and post‐OCR structuring. To guide this analysis, we formulate the following research questions:

1. What are the current methods and pipelines for text detection and recognition applicable to natural history and herbarium documents?
2. How effective are deep learning techniques including convolutional neural networks (CNNs), transformers, vision transformers (ViTs), and large language model (LLM)–based approaches in recognizing printed, handwritten, and mixed‐format text?
3. What datasets are available for OCR and herbarium label digitization, and what are their characteristics and limitations?
4. What challenges specific to herbarium specimens, such as overlapping elements and degraded text, affect the reliability of automated transcription pipelines?

This paper provides a thematic review of methods, datasets, and open problems in automated specimen label processing. We first describe the review methodology, including the search strategy, data sources, scope, and the general architecture of the digitization pipeline. We then present the literature review, covering detection, OCR, and post‐OCR structuring methods. Next, we discuss the datasets used in previous work, examine the advantages and limitations of existing approaches, and outline the associated challenges and open problems. Finally, we conclude with suggestions for directions for future research.

## REVIEW METHODS

### Review protocol

To capture both general developments in text recognition and studies specifically addressing herbarium specimen transcription, a dual search strategy was adopted:

**General search strategy (broad):** This strategy targeted studies on text detection, recognition, and post‐processing across domains relevant to label digitization. The following query was used:

("OCR" OR "text recognition" OR "scene text" OR "handwritten text" OR "HTR" OR "label extraction")

AND

("detection" OR "recognition" OR "post‐processing" OR "extraction")

AND

("CNN" OR "transformer" OR "TrOCR" OR "YOLO" OR "LLM" OR "NER" OR "deep learning")

**Herbarium‐focused search strategy (targeted):** This strategy addressed literature directly concerned with herbarium label processing, including challenges such as varied layouts, handwriting diversity, and specimen overlap. The corresponding query was:

("herbarium" OR "herbaria" OR "specimen sheet" OR "natural history collection")

AND

("label extraction" OR "text detection" OR "transcription" OR "information extraction" OR "OCR")

AND

("deep learning" OR "machine learning" OR "YOLO" OR "CNN" OR "transformer" OR "LLM")

### Data sources

Multiple platforms were consulted to gather relevant literature. Google Scholar provided access to journals, conference papers, and preprints. arXiv enabled the tracking of the latest research developments prior to formal publication. GitHub facilitated the discovery of open‐source repositories, supplementary materials, and relevant datasets, particularly for herbarium and historical documents. Google Search helped identify publicly available OCR datasets and herbarium repositories not indexed in academic databases.

### Study selection and scope

The review considered studies published between 2018 and 2025 that applied deep learning techniques to OCR and handwritten text recognition (HTR) pipelines, post‐OCR structuring, and domain‐specific challenges such as handwriting variability, multilingual labels, and complex layouts. Studies addressing only layout analysis, proprietary OCR benchmarks without methodological contribution, or multilingual OCR unrelated to herbarium documents were excluded.

From an initial set of approximately 80 retrieved articles, titles and abstracts were screened to remove irrelevant studies, followed by full‐text evaluation to assess relevance to the review objectives. Electronic databases and backward reference screening were employed to complement the primary search, ultimately identifying around 50 relevant studies, of which 24 were selected for detailed analysis.

## LITERATURE REVIEW

The general pipeline for automated herbarium label digitization comprises three sequential stages, as illustrated in Figure 2 (De Smedt et al., 2024). The first stage, **label and component detection**, identifies textual and graphical elements on herbarium sheets, including labels, barcodes, and stamps (Thompson et al., 2023; Weaver and Smith, 2023), using preprocessing, segmentation, and object detection techniques to isolate regions of interest from visually complex specimen images. The second stage, **optical character recognition (OCR)**, converts detected text regions into machine‐readable text (Drinkwater et al., 2014); preprocessing steps such as denoising, binarization, and normalization are commonly applied to improve recognition performance. The third stage, **metadata extraction**, parses OCR output into structured fields aligned with Darwin Core standards, including scientific name, collector, collection date, locality, and catalog number, as well as additional taxonomic or geographic information (Wieczorek et al., 2012), producing records that are standardized, searchable, and interoperable for large‐scale biodiversity research. The performance of each stage is evaluated using specialized metrics, summarized in Table 1. The following subsections review existing approaches organized around these three workflow stages (Figure 3).

**Figure 2:**
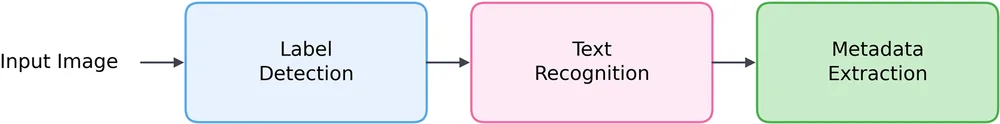
General workflow for digitizing herbarium labels, consisting of detection, recognition, and metadata extraction.

**Table 1:** Performance metrics by pipeline stage.

Metric	Description
Stage: Label and component detection	
Precision	Measures the proportion of detected components that are correct, indicating how many predicted regions correspond to true objects.
Recall	Measures the proportion of true components that were successfully detected, reflecting how many relevant regions the model identifies.
F1‐score	Harmonic mean of precision and recall, useful when balancing false positives and missed detections.
Intersection‐over‐union (IoU)	Computes the overlap between predicted and ground‐truth bounding boxes as the ratio of intersection area over union area.
Mean average precision (mAP)	Summarizes detection quality by averaging precision–recall curves across classes and confidence thresholds.
Stage: Optical character recognition (OCR)	
Character error rate (CER)	Proportion of incorrectly recognized characters relative to the total number of characters; sensitive to small textual errors.
Word error rate (WER)	Measures word‐level mistakes (insertions, deletions, substitutions), better reflecting text usability for downstream tasks.
Stage: Post‐OCR metadata extraction	
Precision	Measures the proportion of correctly extracted metadata fields among all predicted fields.
Recall	Indicates completeness by measuring how many ground‐truth metadata fields were successfully extracted.
F1‐score	Balances precision and recall, providing a single metric to evaluate metadata‐extraction accuracy.
Exact match accuracy	Measures the proportion of records in which every extracted metadata field exactly matches the ground truth.

**Figure 3:**
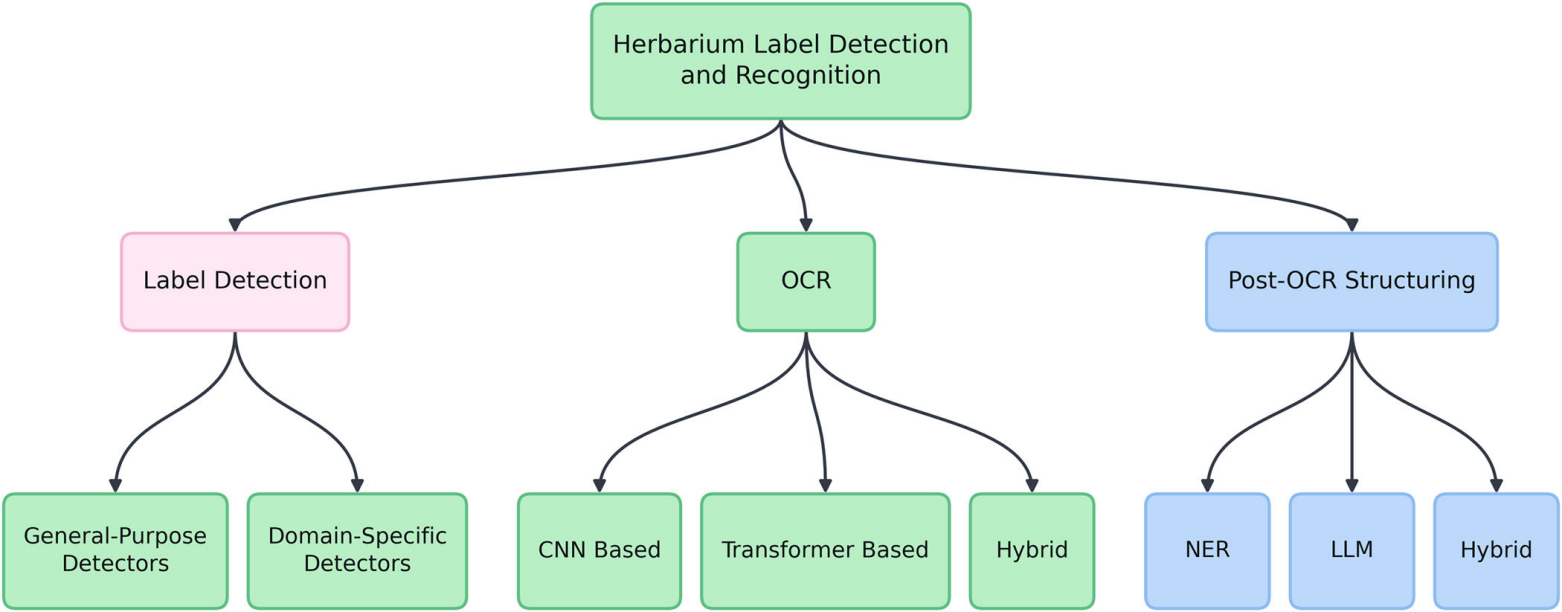
Thematic flow of literature on digitized herbarium label transcription: detection (general and domain‐specific), OCR (CNN, Transformer, hybrid), and post‐OCR structuring (NER, LLM, hybrid).

### Label and component detection

Detecting labels, barcodes, and stamps on herbarium sheets is challenging due to variability in size, orientation, layout, and position, both within and across institutional collections (Figure 1).

#### General‐purpose detectors

Segmentation‐based detectors such as MaX‐DeepLab (Long et al., 2022) and CNN‐based models like DBNet and TextBPN++ (Olejniczak and Šulc, 2023) are widely used in document analysis for text zone detection. These models perform well on structured, high‐resolution documents but are less effective on visually complex layouts. Enhanced DBNet variants with dual‐attention modules (Ding et al., 2024) improve detection in low‐resolution, distorted, and mixed‐language environments, achieving an F‐score of 84.88%, although they remain sensitive to severe distortions. Models such as SwinTextSpotter (Huang et al., 2022) and CRAFT (Baek et al., 2019) target arbitrarily shaped or curved text at the word or sentence level rather than full label regions. For historical or handwritten documents, YOLOv8 (Redmon et al., 2016; Terven and Córdova‐Esparza, 2023) demonstrates strong performance on consistent handwritten layouts, as observed in Scandinavian manuscripts (Marttila et al., 2025). Herbarium sheets, however, combine elements from all these challenges: curved and straight text, mixed fonts, overlapping components, and handwritten labels.

#### Herbarium‐specific detectors

Domain‐specific methods address these challenges more effectively. YOLO is the most frequently used approach when trained on herbarium datasets. YOLOv5 has been applied to detect 11 component types (Thompson et al., 2023), achieving high performance within the training dataset but struggling to generalize across diverse label styles in international collections. The Hespi pipeline (Turnbull et al., 2025) employs a two‐stage YOLOv8 setup: the first stage identifies label types, and the second extracts structured fields such as collector names and dates (Figure 4). This system achieves an F1‐score of 98.5% for institutional labels and a 97.8% mean average precision (mAP) for field‐level components. However, the second stage is limited to a subset of predefined fields and does not capture all available metadata.

**Figure 4:**
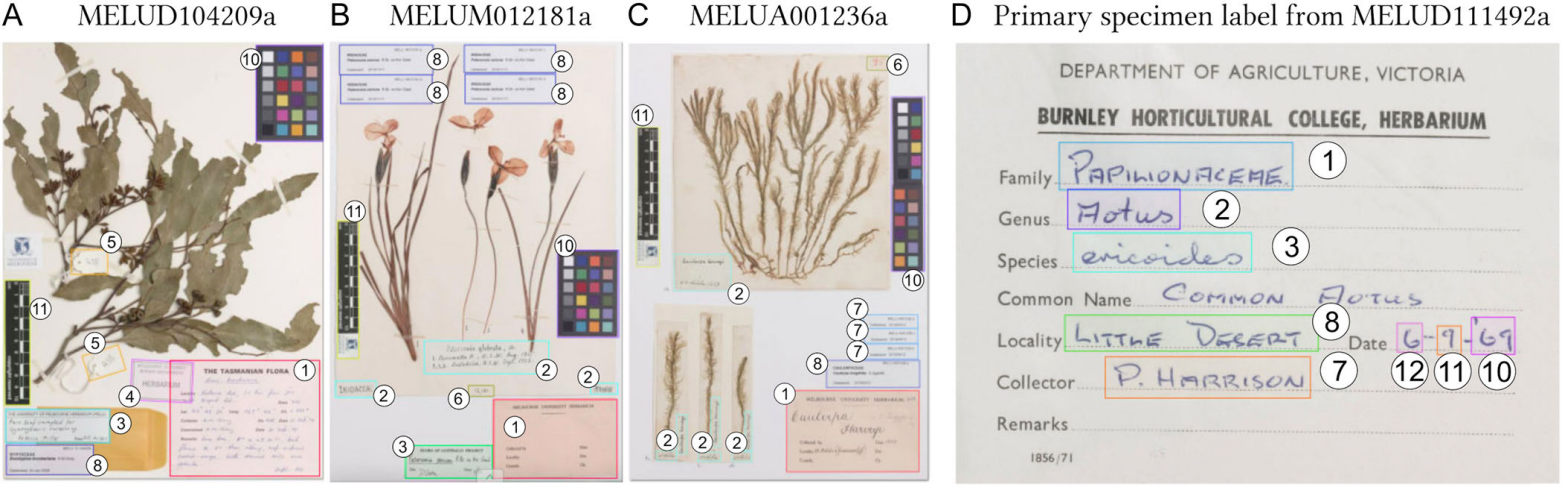
Two‐stage detection in the Hespi pipeline: the first stage (A–C, sheet‐component classes) detects label types and other sheet elements, while the second stage (D, label‐field classes) detects field‐level components such as family, genus, species, collector names, and dates within institutional labels. Numbers correspond to the classes as identified in Turnbull et al. (2025). Reproduced from Turnbull et al. (2025) (CC BY).

### Optical character recognition (OCR)

OCR methods for herbarium labels are grouped below into CNN‐based, transformer‐based, and hybrid approaches, reflecting increasing architectural flexibility.

#### CNN‐based method*s*

CNN‐based approaches rely on convolutional encoders for spatial feature extraction, typically followed by bidirectional long short‐term memory (BiLSTM) layers and connectionist temporal classification (CTC) decoding. Their computational efficiency and ease of deployment make them well suited for relatively structured content such as typewritten labels or consistently formatted handwritten text. CNN‐BiLSTM‐CTC pipelines perform well across multilingual handwritten datasets when text layout is regular. For example, unified CNN–recurrent neural network (RNN) architectures achieved character error rates (CER) of 15.3% on the IAM database (Marti and Bunke, 2002) and 10.0% on the RIMES database (Grosicki et al., 2009; Swaileh et al., 2018). When combined with document text detectors such as DBNet or CRAFT, CNN‐based recognizers have also demonstrated strong performance on structured forms and receipts (Olejniczak and Šulc, 2023), suggesting their applicability to institutional herbarium labels with consistent formatting. In herbarium‐specific workflows, tools such as Tesseract (Smith, 2007) and Google Vision have been widely used for printed and handwritten labels, often in ensemble configurations, achieving reasonable accuracy on curated datasets (Owen et al., 2020). However, their performance remains highly dependent on preprocessing quality and accurate text segmentation, and human validation is still required for handwritten or multilingual content (Weaver et al., 2023; Guralnick et al., 2024b).

#### Transformer‐based methods

Transformer‐based OCR models address limitations related to long‐range dependencies, irregular handwriting, and degraded layouts through attention mechanisms that model contextual relationships across entire text sequences (Li et al., 2021). ViT recognizers have demonstrated strong adaptability to irregular text and multilingual data when fine‐tuned for specific domains (Atienza, 2021). Transformer‐based optical character recognition (TrOCR) achieved low error rates on 19th‐century multilingual manuscripts despite significant handwriting variation (Marttila et al., 2025). Further improvements have been reported through fine‐tuning strategies and low‐rank adaptation methods, which reduce training cost while maintaining accuracy (Hüttner et al., 2025). More recent variants such as TrOCR++ extend this architecture to both handwritten and printed text, showing increased robustness across heterogeneous document types (Rang et al., 2024). However, recognition performance degrades on full‐page or densely populated documents, reflecting ongoing challenges in long‐sequence modeling (Zhang et al., 2023). Transformer‐based models also incur higher computational costs and remain sensitive to input length, making them most effective at the line or word level. Generalization across herbarium collections is further constrained by training data biases and tokenizer limitations, emphasizing the need for domain‐adapted fine‐tuning.

An alternative to multi‐stage pipelines is offered by end‐to‐end multimodal transformers that bypass OCR entirely. Zacchigna et al. (2026) fine‐tuned a document understanding transformer (Donut)–based model (Kim et al., 2022) on herbarium specimens from the University of Pisa, achieving a median tree edit distance (TED) accuracy of 85% on label metadata extraction without requiring a separate OCR step. While this performance remains below that of hybrid OCR‐LLM pipelines, the approach is fully open‐source, operates offline, and eliminates dependencies on commercial APIs, making it well suited for institutions with strict data privacy requirements.

#### Hybrid approaches

Hybrid OCR approaches combine CNN‐based visual feature extraction with transformer‐based sequence modeling to leverage both robust local encoding and flexible attention mechanisms. Models such as SwinTextSpotter and SVTR have achieved high accuracy on benchmark datasets, although robustness decreases under severe distortions or cluttered layouts (Du et al., 2022; Huang et al., 2022). Handwritten‐focused architectures such as HTR‐VT integrate convolutional encoders with ViT blocks and have demonstrated strong performance without relying on large‐scale synthetic data (Li et al., 2025). In the herbarium domain, the Hespi system combines CNN‐based label classification with separate OCR engines (Tesseract for printed text and TrOCR for handwritten content) achieving high accuracy on curated institutional datasets (Turnbull et al., 2025). However, performance decreases on more diverse international collections, highlighting persistent challenges in cross‐collection generalization.

### Post‐OCR structuring

Once text is recognized, it must be organized into structured records aligned with metadata standards such as Darwin Core (Wieczorek et al., 2012; Groom et al., 2019; Engledow, 2022). Three main approaches are used.

1. **NER‐based models:** NER models identify and classify entities within OCR outputs based on predefined categories. They are valued for their interpretability and low computational cost. In general natural language processing applications, spaCy performs effectively with domain‐specific data (Ang et al., 2022). In herbarium contexts, NER can accurately identify entities including numerical data, collector names, and locations. Linking entities to external resources such as the Biodiversity Heritage Library can further improve semantic accuracy (Owen et al., 2020). When applied to Japanese herbarium labels, NER achieves high precision for key fields but still relies heavily on manually curated training data, which may limit scalability (Takano et al., 2024).
2. **LLM‐based structuring:** LLMs can understand context and generate structured outputs, making them flexible for handling varied label formats, multiple languages, and OCR‐noisy text. In historical document applications, models such as GPT‐4o and Claude‐3 achieve high performance not only in entity extraction but also often surpass traditional OCR tools in robustness (Greif et al., 2025). In herbarium digitization, ChatGPT is widely used to correct recognized text, map it to Darwin Core fields, and validate taxonomic names (Weaver et al., 2023; Turnbull et al., 2025). While LLMs can extract more fields than NER, they require human review for complex labels and incur higher costs for scalability. More recently, tools such as SpeciMate (Stenhouse and Thrall, 2025) leverage LLMs through zero‐shot prompt‐based extraction without requiring task‐specific training data, including prompt management systems that enable iterative refinement across projects. Wen (2026) demonstrated that combining PaddleOCR with the DeepSeek LLM for Chinese herbarium labels achieved 95.4% field‐level accuracy on printed labels, with only weak correlation (*r* = 0.32) between OCR confidence and final extraction accuracy, highlighting the compensatory role of LLMs in correcting upstream recognition errors. The integration of LLMs into herbarium digitization pipelines is a recent development: all seven LLM‐involved studies in this review were published between 2023 and 2026, reflecting the rapid adoption of generative AI tools for metadata structuring following the public release of ChatGPT in late 2022.
3. **Hybrid NER** + **LLM approaches:** Some studies combine NER with LLMs to balance precision and flexibility. For instance, ensembles of rule‐based NER and ChatGPT‐4 reduce errors and improve field extraction (Guralnick et al., 2024a). Nevertheless, complex or lengthy labels still benefit from manual verification to ensure completeness.

A comprehensive summary of the works discussed in this review, including pipeline stages, methods, datasets, metrics, and key results, is presented in Table 2. It should be noted that the studies cannot be directly compared based solely on metrics, as each targets specific goals that guide the choice of methods and evaluation criteria.

**Table 2:** Summary of reviewed studies in herbarium and general document analysis.

Reference	Pipeline stage	Approach	Datasets	Metrics	Results	Limitations
Swaileh et al. (2018)	OCR	Bi‐LSTM + CTC, multigram LM	IAM, RIMES	CER, WER	10.0% CER (RIMES), 15.3% CER (IAM)	Older architecture; limited languages
Baek et al. (2019)	Detection	Two‐stage: character region detection + word linking	ICDAR2015	Recall, precision	87.6% recall, 84.3% precision	Less effective for non‐text object types
Owen et al. (2020)	OCR + structuring	OCR + NER on segments	250 herbarium images (Dillen et al., 2019)	Line correctness	77.3% with segmentation	Weak handwritten OCR; incomplete parsing
Atienza (2021)	OCR	Single‐stage STR with ViT encoder	MJSynth, SynthText, IIIT5K, SVT, ICDAR, SVTP, CUTE80	Accuracy	84.2% with data augmentation	Curved/occluded text
Huang et al. (2022)	Detection + OCR	Dilated Swin Transformer, query‐based detector, recognition module	SynthText, ICDAR, Total‐Text, SCUT‐CTW1500	H‐mean, F‐measure	88.0% H‐mean, 74.3% F‐measure	Struggles with long/curved text
Long et al. (2022)	Detection	MaX‐DeepLab for layout + text segmentation	HierText, MLT 2017, Total‐Text, MSRA‐TD500	F‐score, PQ	87.94% F‐score, 53.6 PQ	Limited layout annotations; favors recall
Du et al. (2022)	OCR	CNN + Transformer blocks	MJSynth, SynthText, IC13, IIIT5K, IC15	Accuracy	97.2% (IC13), 86.6% (IC15)	No LM; not robust to distortion
Olejniczak and Šulc (2023)	Detection + OCR	TextBPN++, DCLNet, DBNet with recognizers (CRNN, SAR, MASTER)	FUNSD, CORD, XFUND	F1‐score	96.75% (DBNet + CRNN)	Focus on structured documents
Zhang et al. (2023)	OCR	Growing fine‐tune strategy (TrOCR)	SROIE	F1‐score, CER	87.8% F1, 4.98% CER	Weak on complex layouts
Weaver and Smith (2023)	Detection	YOLOv5x6 ACD model	Herbarium specimens (5573 sheets, 288 herbaria, 101,374 annotations, 9 classes)	mAP@50	0.947 mAP@50 overall, 0.95 for text	Limited to detection; focus on plants rather than labels
Thompson et al. (2023)	Detection	YOLOv5 for label detection	MELU + 9 herbaria datasets	mAP, precision, recall	mAP 0.847, precision 0.983	Poor generalization; retraining needed
Weaver et al. (2023)	OCR + structuring	OCR ensemble + LLM parsing	Herbarium labels dataset	Productivity gain	+25% productivity, 40–60% savings	Requires tuning per institution
Ding et al. (2024)	Detection + OCR	Improved DBNet + CRNN + CTC	ICDAR2015, ICDAR2013	F‐score, precision, recall, accuracy	84.88% detection F‐score, 92.4% recognition accuracy	Sensitive to image distortions
Marttila et al. (2025)	Detection + OCR	TrOCR for HTR + YOLOv8 detection	Historical Scandinavian documents	CER	2.6% CER	Focus on Finnish/Swedish
Li et al. (2025)	OCR	CNN feature extractor + ViT encoder	LAM, IAM, READ2016	CER, WER	2.8% CER, 7.4% WER	No pretraining; no language model
Rang et al. (2024)	OCR	TrOCR++ encoder‐decoder	IAM, IIIT5K, SVT, CUTE, ICDAR, DocVQA	F1, CER, accuracy	IAM: 8.6% CER, IIIT5K: 94.3%	Limitations in full‐page OCR
Guralnick et al. (2024b)	Detection + OCR	YOLOv7 + OCR ensemble + human validation	Herbarium sheets	Accuracy, character error	93.9% accuracy, 4× error reduction	Needs human input; English only
Takano et al. (2024)	OCR + structuring	OCR + NER (BERT, ALBERT, spaCy)	Japanese herbarium labels	F‐score	spaCy highest F‐score	Manual annotation; Japanese only
Hüttner et al. (2025)	OCR	TrOCR + LoRA/DoRA	IAM, READ2016	CER, WER	DoRA: 4.45% CER, FT: 5.97% CER	No augmentation; English only
Greif et al. (2025)	OCR + structuring	Multimodal LLMs for OCR, correction, NER	120 German documents, 5000+ entities	CER, accuracy, F1	GPT‐4o and Mixtral outperform TrOCR	High compute; prompt dependency
Kanerva et al. (2025)	Structuring	LLMs for OCR post‐correction	ECCO‐TCP, National Library of Finland	CER, WER	76.5% CER improvement	Poor low‐resource language handling
Guralnick et al. (2024a)	Structuring	Rule‐based + ChatGPT‐4	2128 herbarium labels	Error count, quality	68 errors vs. 193 baseline; 50% error‐free	Needs validation; long labels degrade performance
Turnbull et al. (2025)	Detection + OCR + structuring	YOLOv8 + Tesseract + TrOCR + GPT‐4o	4821 MELU + herbarium datasets	F1, mAP@50, similarity	98.5% F1, 95.9% similarity	Limited generalization; high resource usage
Ang et al. (2022)	OCR + structuring	Tesseract OCR + spaCy + Med‐7 NER	Philippine prescriptions	CER, WER, F1	4.55% CER, F1: 0.9316	OCR errors; NER splitting issues
Wen (2026)	OCR + structuring	PaddleOCR + DeepSeek LLM	600 Chinese herbarium specimens (3 herbaria: PE, KUN, IBSC)	Field‐level accuracy	95.4% printed, 64.7% handwritten	Handwritten performance limited; Chinese labels only
Zacchigna et al. (2026)	OCR + structuring	Donut (end‐to‐end multimodal Transformer)	45,951 herbarium specimens (University of Pisa)	TED accuracy	85.1% median TED accuracy	Multi‐label sheets; handwriting challenges; limited metadata fields

*Note*: CER, character error rate; CNN, convolutional neural network; CTC, connectionist temporal classification; HTR, handwritten text recognition; LLM, large language model; mAP, mean average precision; NER, named entity recognition; OCR, optical character recognition; STR, scene text recognition; TED, tree edit distance; ViT, vision transformer; WER, word error rate.

## DATASETS

The diversity of training and evaluation data directly influences the generalizability of detection, OCR, and structuring models. This is particularly relevant for herbarium labels, whose visual complexity demands datasets that capture a wide range of text styles, layouts, and conditions.

A large number of general OCR datasets are publicly available and primarily designed for document or scene text recognition; however, handwritten and mixed‐content scenarios remain underrepresented, limiting the transferability of models trained on these benchmarks to botanical labels. Only a small fraction of datasets are specifically tailored to herbarium collections, and these typically focus on label detection, field classification, or structured data validation rather than full‐text recognition. Moreover, most herbarium‐specific datasets are derived from single institutional collections, constraining their applicability across multiple herbaria. To date, no publicly available dataset directly targets OCR or NER training on full herbarium labels, representing a critical gap for end‐to‐end digitization pipelines. Similar annotation challenges have been reported for phenological trait analysis, where deep learning approaches require large volumes of expert‐labeled data across diverse floras (Lorieul et al., 2019). Representative OCR and herbarium datasets, including text types, domains, sizes, annotations, and primary use cases, are summarized in Table 3. General OCR datasets collectively provide two orders of magnitude more annotated samples than the available herbarium‐specific datasets, none of which include line‐level OCR annotations or entity‐level NER labels suitable for training text recognition or information extraction models. Expanding the availability of larger, standardized, and multi‐institutional datasets that jointly support detection, OCR, and field extraction remains essential for reproducible evaluation and robust model development.

**Table 3:** Representative OCR and herbarium datasets with task usage.

Datasets	Text type	Domain	Size	Annotations	Use case/task
General OCR datasets					
ICDAR2015 (Karatzas et al., 2015)	Printed	Scene text	1500+ images	Word‐level bounding boxes	Text detection and recognition
IIIT5K (Mishra et al., 2012)	Printed	Scene text	5000 words	Word‐level annotations	Scene text recognition
SynthText (Gupta et al., 2016)	Synthetic	Scene text	800,000+ images	Word‐ and character‐level annotations	Pretraining OCR models
IAM handwriting (Marti and Bunke, 2002)	Handwritten	English documents	1539 pages	Line, word, character labels	Handwriting recognition
RIMES (Grosicki et al., 2009)	Handwritten	French documents	12,723 forms	Line‐ and word‐level annotations	Handwriting recognition in French
RVL‐CDIP	Printed	Scanned documents	400,000+ pages	Document‐level labels (16 classes)	Document classification and layout analysis
Herbarium‐specific datasets					
MELU label classification (Turnbull et al., 2024a)	Mixed	Herbarium sheets	3152 images	Label type + train/validation split	Label style classification (handwritten, printed, typewritten)
MELU component detection (Thompson and Turnbull, 2023)	Mixed	Herbarium sheets	3371 images	YOLO object annotations for labels	Component detection
MELU field detection (Turnbull et al., 2024b)	Mixed	Institutional labels	3642 images	YOLO‐format bounding boxes for 12 semantic fields	Metadata extraction (genus, species, locality)
DILLEN benchmark (Dillen et al., 2019)	Mixed	Herbarium sheets	1800 images	Full transcriptions for printed and handwritten labels	OCR evaluation, handwriting recognition, label segmentation
Digi‐Leap GBIF subset (Denslow, 2024)	Printed	GBIF metadata	2000 records	Metadata with scoring outputs	Evaluation of digitization and transcription workflows

## DISCUSSION

Notably, among the 26 studies reviewed (Table 2), only Hespi (Turnbull et al., 2025) integrates all three pipeline stages within a single framework. Most studies address one or at most two stages in isolation, which limits end‐to‐end performance evaluation and complicates cross‐study comparison. Half of the reviewed studies (13 out of 26) focus on a single pipeline stage, predominantly OCR (seven studies), reflecting the field's historical emphasis on text recognition as a standalone task rather than as a component of an integrated digitization workflow.

YOLO‐based detectors demonstrated strong object‐level performance on domain‐specific herbarium datasets, but this success depends on large, annotated training sets and can lead to overfitting to the characteristics of specific collections (Thompson et al., 2023; Guralnick et al., 2024b). Two‐stage detection pipelines such as Hespi (Turnbull et al., 2025) achieve high accuracy for essential Darwin Core fields but do not perform full‐text coverage, potentially omitting contextual information outside the targeted fields. A hybrid detection framework may address this limitation by first localizing key regions using object detectors (e.g., YOLO) and then applying fine‐grained character‐aware models such as CRAFT within these areas. This approach leverages the high recall of detection models alongside the precision of text‐specific segmentation, offering a scalable, modular pipeline for diverse specimens. Moreover, the two herbarium‐specific detection studies (Thompson et al., 2023; Weaver and Smith, 2023) focus exclusively on component localization without integrating a downstream OCR or structuring module, leaving the question of how detection quality propagates through the full pipeline largely unaddressed.

The quality and resolution of digitized images also affect pipeline performance. Phang et al. (2022) demonstrated that even high‐resolution digital images may fail to capture diagnostic micro‐morphological characters, a limitation that extends to automated text recognition when labels are degraded, faded, or partially obscured by plant material. For OCR, hybrid approaches such as Hespi effectively combine models optimized for printed and handwritten content but require a pre‐classification step that introduces additional complexity and potential error propagation. Transformer‐based recognizers such as TrOCR provide flexibility in handling heterogeneous content without separate models, provided sufficient domain‐specific training data are available, while CNN‐based approaches such as CRNN (Owen et al., 2020; Olejniczak and Šulc, 2023; Guralnick et al., 2024b) remain computationally efficient on clean printed labels. At the pipeline level, the most common combination among herbarium‐specific studies is OCR paired with structuring (five out of 10 studies), typically assuming that text regions have already been identified or manually cropped. This pattern highlights a disconnect between the detection and downstream stages, as the influence of detection errors on OCR and extraction accuracy remains largely unexplored.

For structuring OCR output, ensemble approaches (Guralnick et al., 2024a) that combine high‐precision NER with the contextual flexibility of LLMs can improve both accuracy and adaptability. In low‐resource settings targeting only key Darwin Core fields, NER‐based pipelines (Takano et al., 2024) remain effective when sufficient annotated data are available. LLM‐only pipelines (Weaver et al., 2023) are suitable for rapid prototyping; however, their integration introduces methodological considerations including non‐deterministic outputs, prompt sensitivity, and potential model drift, which require additional validation. Beyond metadata structuring, LLMs have also demonstrated potential for downstream tasks: Xie et al. (2025) showed that GPT‐4o can perform georeferencing of herbarium specimen labels with near‐human accuracy (median error < 10 km) at a fraction of the time and cost of manual methods, suggesting that LLM‐based approaches may extend well beyond OCR post‐correction to encompass the full chain of information retrieval from specimen labels.

Interestingly, the structuring stage shows the strongest domain specificity: seven of the 10 studies addressing metadata extraction target herbarium collections directly, compared to only four of 10 for detection. This asymmetry suggests that while detection methods can be adapted from general computer vision, the structuring of OCR output into Darwin Core fields remains a challenge inherently tied to the herbarium domain.

Cost and deployment considerations further shape these decisions. Commercial generative AI services demonstrate strong OCR and parsing capabilities but introduce recurring costs, external dependencies, and data governance constraints. LLM‐based extraction tools can harmonize heterogeneous collections at scale but may also increase fragmentation through custom prompts, proprietary models, and institution‐specific workflows. Institution‐level performance differences, such as those reported by Wen (2026) across three Chinese herbaria using identical pipelines, suggest that institution‐specific prompt customization may become common practice, risking further workflow fragmentation across the global digitization community. Some recent approaches address data security by design: Wen (2026) processes specimen images locally and sends only extracted text to the LLM API, while Zacchigna et al. (2026) demonstrated a fully open‐source, locally deployable, end‐to‐end Transformer model that requires no external services, eliminating data privacy concerns but currently at the cost of lower extraction accuracy. Tools such as SpeciMate (Stenhouse and Thrall, 2025) support both commercial API‐based and locally hosted LLMs, although local deployment currently yields lower quality and speed. The optimal choice therefore depends on available resources, performance requirements, and long‐term sustainability considerations, including scalability, maintenance, and domain adaptation.

## CHALLENGES AND OPEN PROBLEMS

Despite recent progress, several challenges remain that go beyond the method‐level limitations discussed above. We organize these challenges around four axes: linguistic complexity, LLM reliability, data governance, and ecosystem fragmentation.

### Linguistic and semantic variability

Herbarium labels combine Latin nomenclature, vernacular and indigenous terms, historical abbreviations, and inconsistent field terminology, often within a single sheet. These factors require models to generalize across languages, dating conventions, and domain‐specific formats, a challenge that current datasets, predominantly drawn from single institutional collections, are insufficient to address.

### LLM reproducibility and prompt sensitivity

The integration of LLMs into digitization pipelines introduces methodological concerns that have no equivalent in traditional OCR workflows. Non‐deterministic outputs mean that identical inputs may yield different results depending on sampling strategies, internal model states, or subsequent model updates (Atil et al., 2025). This variability is compounded by sensitivity to prompt design: domain‐specific prompt strategies are often required to handle the conventions of particular collections. For instance, Wen (2026) developed five prompting principles for Chinese herbarium labels, including field alias normalization and contextual association, achieving 95.4% field‐level accuracy on printed labels. However, these prompts were tailored to Chinese label conventions and would require substantial adaptation for collections using different languages, dating systems, or layout standards. Model drift further compounds these issues, as LLM updates over time may cause identical workflows to generate divergent outputs across different stages of a long‐running digitization project. Addressing these concerns requires careful documentation of prompts, model versions, and extraction workflows, alongside validation protocols and consensus mechanisms.

### Data governance and sensitive information

Herbarium labels may contain precise locality data for threatened or endangered species. Automated extraction at scale requires consideration of data sharing policies, including compliance with frameworks such as the CARE principles for indigenous data sovereignty (Carroll et al., 2020) and relevant data protection regulations. The architectural choices discussed in the Discussion section (commercial APIs, locally deployed models, or end‐to‐end offline systems) each carry different implications for data governance, and no consensus has emerged on best practices.

### Ecosystem fragmentation

The proliferation of institution‐specific prompts, proprietary models, and divergent extraction configurations risks creating a landscape in which the same specimen yields different structured outputs depending on the tools used. Rather than converging toward interoperable standards, the flexibility of LLM‐based approaches may paradoxically increase heterogeneity across the global digitization community. Mitigating this risk will require shared prompt registries, community‐agreed validation benchmarks, and coordinated governance frameworks that support both standardization and institutional autonomy.

## CONCLUSIONS

This review addressed the four research questions posed in the Introduction. Regarding detection methods (Question 1), YOLO‐based approaches dominate herbarium‐specific applications, with two‐stage pipelines such as Hespi achieving high field‐level accuracy, although full‐text coverage remains unaddressed. For OCR effectiveness (Question 2), transformer‐based models such as TrOCR outperform CNN‐only pipelines on mixed‐content labels, while hybrid architectures offer practical compromises between accuracy and computational cost; in both cases, domain‐specific fine‐tuning remains essential. The dataset landscape (Question 3) reveals a critical gap: no publicly available benchmark directly targets OCR or NER training on full herbarium labels. Finally, persistent challenges (Question 4) related to layout variability, LLM reproducibility, prompt sensitivity, and data governance underscore the need for modular, well‐documented, and reproducible pipelines.

Looking ahead, progress will depend on three complementary efforts: (1) the development of unified, adaptable pipelines that integrate semi‐supervised learning, active annotation, and domain‐tuned foundation models to reduce reliance on large manually curated datasets; (2) the establishment of community‐shared resources (including standardized benchmarks, prompt registries, and validation protocols) to support reproducibility and interoperability across institutions; and (3) coordinated governance frameworks that address both data sensitivity concerns and the risk of ecosystem fragmentation arising from the proliferation of institution‐specific workflows. Together, these efforts would enable herbarium collections to be more fully exploited for biodiversity research, from species distribution modeling and phenological analysis to functional trait studies at global scales.

## AUTHOR CONTRIBUTIONS

H. Abbad conceived the study, carried out the literature investigation and analysis, prepared the figures, and wrote the original draft of the manuscript. Y.S. supervised and administered the project, acquired funding and resources, contributed to validation, and revised the manuscript. E.C., E.P., and J.‐D.Z. acquired funding and resources and revised the manuscript. N.T. contributed to validation and revised the manuscript. H. Ariouat and A.S. revised the manuscript. All authors approved the final version of the manuscript.
